# Nutritional Cues Tie Living Organisms to Their Environment and Its Sustainability

**DOI:** 10.3389/fnut.2016.00028

**Published:** 2016-08-12

**Authors:** Melanie S. Adams, Robert B. Adams, Carol A. Wessman, Barbara Demmig-Adams

**Affiliations:** ^1^Department of Anthropology, University of Colorado, Boulder, CO, USA; ^2^Department of Ecology and Evolutionary Biology, University of Colorado, Boulder, CO, USA; ^3^Cooperative Institute for Research in Environmental Science, University of Colorado, Boulder, CO, USA

**Keywords:** inflammation, antioxidants, agriculture, fatty acids, glycemic, obesity, crop yield, crop stress tolerance

## Abstract

We connect modern, intensive agriculture’s role in environmental degradation to its role in producing nutritionally unbalanced foods, and delineate specific approaches to reduce agriculture’s environmental impact, while producing healthful foods. We call attention to recently discovered genetic programs used by all living organisms to respond to their environment, and present a model of how these programs change body composition and function (of humans and their crop plants and livestock alike) in response to environmental cues. We propose that production of nutritionally balanced crops and livestock requires careful consideration of how these plants and animals are grown; the composition of plant food is modulated by growing conditions, body composition of livestock reflects their feed; composition and function of human body and brain are strongly affected by how food plants and animals are produced. We selected four nutritional features not only involved in (i) governing human health by modulating these genetic programs, but (ii) also affected by agricultural practices. These nutritional features are fat composition (especially saturated fat and the ratio of polyunsaturated omega-6 oils to omega-3 oils), carbohydrate composition (especially the proportion of carbohydrates with a high glycemic index, such as sugars and quick-burning starches) and the level of antioxidant micronutrients. We not only outline threats to human health presented by the current environment, but also potential gains in quality-of-life in a future environment designed to optimize human wellness using insights into the gene-programing effect of diet- and other lifestyle-related factors. These gains could extend beyond optimal human physical and mental health to gains in workforce productivity. The same changes in agricultural practices required to achieve these gains in human health are also needed to support environmental health and sustainable food production. The resulting vision of optimal human health and environmental health, supported by sustainable practices, is intended as an inspiring image of what sustainability has to offer to individuals and society. Our goal is to provide a transparent overview and illustrations intelligible not only to non-experts in each of the other respective areas involved but also to policy makers and the public.

## Introduction

Pressing concerns of society are frequently debated as separate entities: preserving the rainforests, eradicating cancer and diabetes, and feeding starving children in Africa. Does humanity have to choose between fostering either human health or environmental protection – or can we accomplish both, while simultaneously serving social equity by feeding the proliferating human population? Recent analyses indicate that significant increases in food production are possible, and that the challenge will indeed be to achieve this increase in a sustainable, equitable manner (1, 2). The present paper evaluates how nutritious food that fosters human health can be produced in an environmentally sustainable and equitable way.

Through integration of recently discovered mechanistic relationships between agriculture and human wellness, we hope to connect the conversation about environmental sustainability to specific features of human diseases and disorders, thus making environmental issues more personally relevant to large constituencies. Despite urgent recommendations from environmental scientists to invest in more sustainable approaches to modern living, decisive action by policy makers and the public has yet to be taken at a sufficient level to address unprecedented environmental degradation, exhaustion of natural resources, and climate change (3). So far, many authors have encouraged making connections between environmental degradation, economic interests, and social equity to increase motivation among non-experts for wide-scale engagement with environmental issues (4, 5). Specifically, it has been proposed that true sustainable development must address three major areas: environment, economy, and society including social equity (6–8). Increasingly, attention is also being given to connections to human health and nutrition (9–12). Rather than attempting to comprehensively review each area, this overview makes selected connections among human nutrition, human physiology and evolution, environmental and climate science, and agricultural science, in the context of sustainability. We deliberately aim for transparency over exhaustive detail in the hope that our synthesis will be intelligible and inspiring to not only non-experts in each of the other respective areas touched upon, but also to policy makers and the public. In addition to highlighting links between nutrition and sustainability, we aim to enhance the non-expert’s motivation for following dietary guidelines by highlighting selected examples of how diet governs mental and physical health. The illustrations in this overview are designed to provide transparent information suitable for use by non-experts and in consultations among constituents with widely different backgrounds.

It has been demonstrated that modern, intensive agriculture is a major contributor to environmental degradation, depletion of natural resources, and climate change, which, in turn, increasingly threatens future food security through drought, extreme weather events, and epidemics of crop pests and diseases (1, 13–15). Other authors have posited that the food produced by modern, intensive agriculture – what is grown and how it is grown – is nutritionally deficient and contributes to the risk for human disease (9, 10, 12). Tilman and Clark (12) called for an elucidation of mechanisms underlying these links.

**Figure 1:**
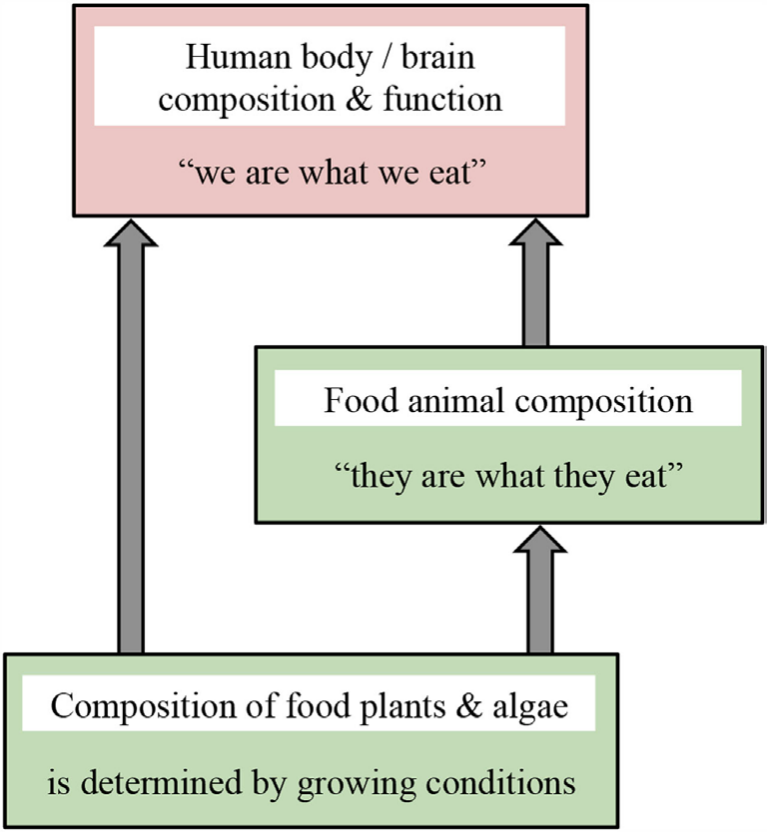
**Schematic depiction of the message that growing conditions or what is eaten strongly impacts composition and function of living organisms**. Crop composition is modulated by factors such as water and nutrient supply, body composition of livestock reflects their feed, and composition and function of human body and brain are determined by what we eat.

We here call attention to recently discovered genetic programs used by all living organisms to respond to their environment. We provide illustrative examples of how these programs change body composition and function in response to environmental cues, thereby allowing organisms to cope with natural fluctuations in their environment. We relate these responses to agriculture and evaluate how agricultural practices affect key nutritional features that govern human health. Figure 1 summarizes our central message that the composition of food plants/crops is modulated by growing conditions, such as water and nutrient supply; body composition of food animals/livestock reflects their feed (“they are what they eat”); and composition and function of human body and brain are determined by what we eat (“we are what we eat”). Therefore, production of nutritionally balanced crops and livestock requires careful consideration of how food plants and animals are grown. However, modern intensive agricultural practices were designed before these genetic programs, and their modulation by environmental conditions including diet, had been recognized. By causing fundamental changes in the composition of food plants and animals compared to the foods humans evolved with, current agricultural approaches increase the risk for human diseases and disorders. We demonstrate how knowledge of specific dietary requirements for human health can be utilized to design food production systems that not only meet nutritional needs but also do so in a sustainable manner while producing enough food to support the proliferating world population. Specifically, we identify:

- four selected nutritional features that play key roles in the control of human body composition and function.
- how imbalances in these nutritional features can lead to chronic human diseases as well as mental and learning disorders, while balanced intake of these four nutritional factors promises to promote human wellness.
- the mechanisms by which these four nutritional features orchestrate critical body functions like fat storage and the immune response.
- the impact of crop growth and livestock rearing conditions on food nutritional quality with respect to these four key nutritional factors.
- how this knowledge can aid in the design of food production systems that simultaneously promote human wellness, preserve natural resources, and feed the world population.

## Overview of Links Between Diet and Human Health: Four Key Dietary Features

Nutritional imbalances increase human susceptibility to both chronic disease and infectious disease [see Ref. (16) for a review on the link to infectious disease]. Here, we focus on the effect of diet on non-communicable, chronic diseases (such as heart disease, cancer, diabetes, neurodegenerative diseases like Alzheimer’s or Parkinson’s, and others) as well as mental and learning disorders (such as depression, schizophrenia, attention deficit, autism, and others). Non-communicable diseases and disorders “are predicted to cost the global community more than US $30 trillion over the next 20 years” (17).

All countries today are at some stage of a nutrition and lifestyle transition, characterized by a combination of reduced physical activity with increased consumption of sugary drinks and highly processed, sugar-rich foods [(18, 19); see also Ref. (17)]. Nutritionally unbalanced foods contribute heavily to epidemics of chronic disease and disorders as well as widespread disability and early death (9, 17, 18). Unbalanced diets typically provide not only too much sugar and saturated fat but also insufficient levels of omega-3 fatty acids and micronutrients, among which are vitamins and antioxidants (see definitions in Table 1). Here, we introduce a model of selected mechanisms that illustrate links between nutritional imbalances and (i) chronic diseases and disorders on one hand as well as (ii) crop and livestock production conditions of modern, high-input agriculture on the other hand. While personal choice may help avoid nutritionally unbalanced, highly processed foods, system-wide changes in agricultural and other practices are called for to fully support human wellness (9, 11, 20). While a range of dietary nutrients is known to be required for human health [see Ref. (21)], we call attention to recently discovered genetic programs used by living organisms to respond to their environment. We provide illustrative examples of how these programs change body composition and function in response to environmental cues, thereby allowing organisms to cope with natural fluctuations in their environment.

**Table 1:** **Definition or description and dietary sources of four key nutritional features required in balanced levels to lower the risk for chronic human diseases and disorders: (i) antioxidants, (ii) glycemic load and glycemic index, as well as fat composition, i.e., (iii) the ratio of omega-6 to omega-3 polyunsaturated fatty acids, and (iv) the level of saturated fat**.

**Figure 2:**
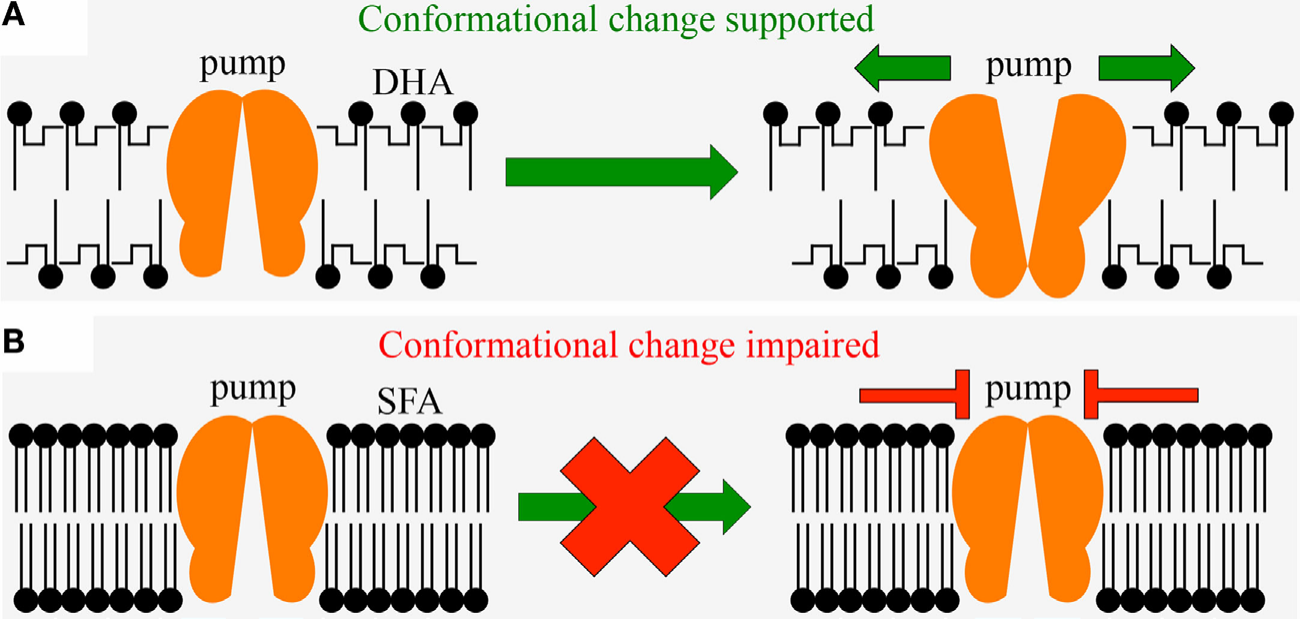
**Diagram showing the effect of the fatty acid composition of membrane phospholipids on the fluidity of biological membranes**. Membranes need to be fluid enough to allow the sodium–potassium pump (orange) to undergo shape changes (conformational changes) underlying the pump’s ability to generate the chemical and electrical potential across the membrane required for nerve impulses as critical functions of the brain and the heart muscle. **(A)** sufficient availability of the polyunsaturated fatty acid DHA (with highly bent fatty acid tails that result in loose, fluid membranes) in the diet is required to support the pump’s conformational change; **(B)** excessive consumption of saturated fat with its straight, rigid fatty acid tails (SFA) that result in tightly packed membranes, impairs ability of the pump to undergo conformational change. Since polyunsaturated fatty acids are vulnerable to oxidation, sufficient levels of antioxidants are required to maintain the integrity of DHA and its function in nerve impulses. DHA, docosahexaenoic acid (a polyunsaturated omega-3 fatty acid); SFA, saturated fatty acid.

**Figure 3:**
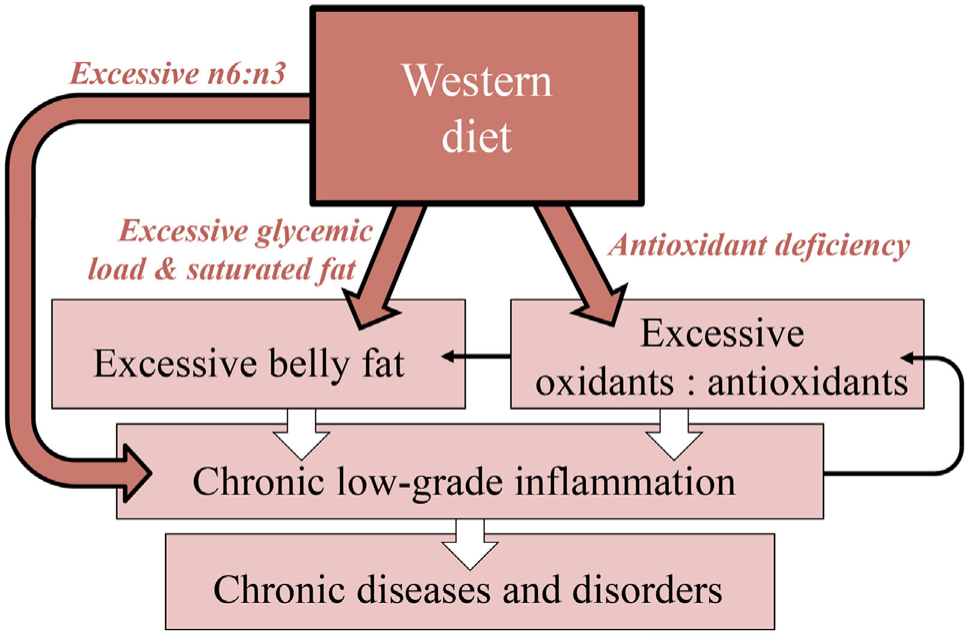
**Diagram of the synergistic interaction among key nutritional features of the Western diet in promoting chronic diseases and disorders**. Highlighted are the mechanistic links of how these factors promote excessive belly fat, and how this belly fat, together with excessively high ratios of omega-6 to omega-3 fatty acids and of oxidants to antioxidants, trigger chronic low-grade inflammation (wide arrows). Thin arrows indicate additional feed-forward loops between these processes [see, e.g., Ref. (56)]. *n*6:*n*3, ratio of omega-6:omega-3 fatty acids; GL, glycemic load; SF, saturated fat, AO, antioxidants.

**Antioxidants**
Antioxidants balance the action of oxidants. Oxidants are reactive molecules that can damage various biologically important molecules in the human body by oxidation (extraction of electrons), which sets off a chain reaction by creating radicals and other reactive molecules (22). Antioxidants prevent the oxidative damage of other molecules by donating an electron while remaining stable (22). On the other hand, small amounts of oxidants are needed to trigger vital body responses, and a balanced ratio of oxidants and antioxidants is thus needed to support wellness (see also Figure 3 below). Dietary antioxidants include the vitamins E and C and many other plant chemicals found in vegetables, fruits, herbs, and spices (23, 24).
**Glycemic load and glycemic index**
Glycemic index quantifies how quickly a carbohydrate-based food is broken down into free sugars, which may drive up blood sugar levels, causing hyperglycemia. Glycemic load is the product of the glycemic index for a food and the amount of that food consumed. A diet providing an excessively high glycemic load increases obesity and the risk for chronic diseases and disorders (see also Figure 3 below). Sweets, white bread, white rice, and baking potatoes are examples of foods with a high glycemic index; whole-grain products, many vegetables, and most animal products have a low glycemic index.
**Essential omega-3 and omega-6 fatty acids**
Derivatives of polyunsaturated fatty acids function as body constituents needed in particularly high supply in the brain for mental function (Figure 2) and in the heart muscle for a regular heartbeat. These fatty acids also serve as precursors for hormone-like modulators of human metabolism (see Figure 3). Omega-3 and omega-6 oils must be consumed in balanced ratios to avoid derailing human metabolism (see below). Vegetable oils like corn, sunflower, soybean, and cottonseed oil have excessively high levels of omega-6 oils (25); cold-water fish, eggs, and nuts can be good sources of omega-3 oils if grown/raised appropriately (25–27).
**Saturated fat**
Excessive consumption of saturated fat increases obesity and the risk for chronic diseases and disorders, just as is the case for a diet providing a high glycemic load (see also Figure 3 below). A balanced diet should be low in saturated fat. Saturated fat is found primarily in animal sources. Meat from physically inactive livestock raised on high-calorie supplemental feed tends to be excessively high in saturated fat.

Table 1 lists and defines four key nutritional features that are required in balanced levels to lower the risk for multiple human diseases and disorders: (i) antioxidants, (ii) glycemic load and glycemic index, as well as fat composition, i.e., (iii) the ratio of omega-6 to omega-3 polyunsaturated fatty acids and (iv) the level of saturated fat. Some of these factors (polyunsaturated fatty acids, antioxidant metabolites, and minerals needed as cofactors for antioxidant enzymes) are essential nutrients required by humans that must be consumed through the diet because they cannot be synthesized in the human body (28). As will be elaborated below, these diet-derived factors can serve as body constituents and/or regulate important body functions. It is important to realize that it is not possible to recommend reliable sources of, for example, diet-derived omega-3 fatty acids without specifying the food-production conditions. As a prime example, fish is only a source of omega-3-rich “fish oils” if the fish has consumed algae and/or other microorganisms that manufacture these oils (29, 30). As another example, eggs can be a rich source of omega-3 fatty acids, as is the case for free-ranging chickens (31) that presumably had access to these fatty acids in their own diet.

## Mechanistic Links Between Key Nutritional Features and Human Diseases or Disorders: Body Constituents

An important role of the human diet is to provide essential body constituents. For example, the omega-3 fatty acid docosahexaenoic acid (DHA) is a critical component of the human brain; DHA should constitute 25% of the brain’s gray matter (32). A combination of omega-3 fatty acids and antioxidants is required for producing and maintaining brain tissue and nerve function for mental acuity and to help prevent mood and learning disorders. Both DHA and antioxidants impact, for example, the function of the sodium–potassium pump, a major brain protein that establishes the conditions required for generation of nerve impulses [Figure 2 (22, 33–35)]. Only the bent, highly flexible shape of DHA – as opposed to the straight, rigid shape of saturated fatty acids – allows the shape changes that underlie the sodium–potassium pump’s action (22), which is prerequisite for nerve action potentials as the basis for human thought processes (Figure 2). The flexible structure of DHA is due to its many unsaturated chemical bonds, which also makes DHA highly susceptible to oxidation (22). Antioxidants are thus needed to detoxify oxidants and preserve the function of DHA and of the sodium–potassium pump (22). Mental disorders, learning disorders, and mood disorders have all been linked to dietary deficiencies [for reviews, see Ref. (17, 36)] in antioxidants (37–39) and omega-3 fatty acids [for both nutrients, see Ref. (22, 34)]. Specifically, omega-3 fatty acid and antioxidant deficiency is thought to be involved in attention deficit hyperactivity disorder, depression, autism, schizophrenia, and neurodegenerative diseases (22, 34) such as Alzheimer’s and Parkinson’s (28). In light of the mechanisms linking diet to brain composition and the generation of nerve impulses, it comes as no surprise that an unbalanced diet impairs critical mental capabilities such as attention, reasoning, judgment, memory, and mood stability.

Several clinical trials have established causal links between human mental acuity and/or violent behavior and dietary deficiencies in omega-3 oils and antioxidants [e.g., Ref. (36, 40, 41)]. A combination supplement containing both omega-3 oils and antioxidants produced a significant gain in academic performance (42) and reductions in antisocial and violent behavior in school children (40). Another study (41) demonstrated statistically significant improvements in performance and behavior through the administration of large doses of mineral and vitamin supplements to children with bipolar disorder or severe attention deficit. However, other studies on this topic had different outcomes. Several comprehensive reviews of all clinical trials performed with either omega-3 fatty acid, antioxidant, or amino acid supplements reported mixed results (43, 44). This outcome may be due to the fact that further nutritional features, in addition to omega-3 oils and antioxidants, are important for brain function. In addition to effects of yet other nutritional factors, all four nutritional features listed in Table 1 contribute toward maintaining balanced conditions in brain and body, as will be described in the next section for the example of the regulation of the immune system.

The traditional Mediterranean diet possesses all four nutritional features in the proportions that lower disease risk; it provides a low glycemic load, is low in saturated fat, and rich in omega-3 fatty acids and antioxidants [see Ref. (22, 45)]. In contrast, the modern Western diet, and even vegetarian and/or fish-based diets, can be nutritionally unbalanced (see section on “Vegetarian Diets” below). In a groundbreaking clinical trial with thousands of participants who had suffered an early heart attack, a traditional Mediterranean diet dramatically lowered (by 72% over 5 years) the risk of a second heart attack (46). The Mediterranean diet also produced dramatic health benefits in studies of clinical depression (47), Alzheimer’s (17, 48), diabetes (49), and other chronic conditions (50).

## Mechanistic Links Between Four Key Nutritional Features and Human Diseases or Disorders: Immune System Regulation

Modulation of the immune system is an example for how the four key dietary factors introduced in Table 1 interact synergistically to affect physical and mental health. Figure 3 introduces an integrative scheme, in which we synthesize a series of recent discoveries, of how antioxidants, glycemic load, saturated fat content, and omega-6 to omega-3 fatty acid ratio contribute to the modulation of inflammation as a key function of the immune system. A continuously activated immune system causes immune cells to mount a state of low-grade chronic inflammation, wherein white blood cells attack uninjured body cells (51, 52). Most of today’s chronic human health problems have been demonstrated to be pro-inflammatory diseases and disorders, involving chronic, low-grade inflammation, in which an overactive immune system attacks the body’s own nerve cells, heart cells, insulin-producing cells, and other cells, thus contributing to neurodegenerative diseases and heart disease (53), diabetes, autism, depression, schizophrenia, attention deficit, and other conditions (17, 22, 54, 55).

Under the following sub-headers, we describe our model of how inflammation is stimulated by excessive belly fat (visceral fat), which is, in turn, promoted by excessive glycemic load and saturated fat, by an excessive ratio of oxidants to antioxidants (57, 58), and by an excessive ratio of omega-6 to omega-3 fatty acids (9, 27, 59, 60).

### Diets High in Glycemic Load and Saturated Fat Promote Excess Fat Storage and Chronic Inflammation

The human immune response requires a considerable energy investment and has apparently evolved to adjust the defense response to the energy available for expenditure (61, 62). In fact, the human immune system is so tightly linked to available energy stores that it is “often up-regulated by nutrition-related signals, independent of the actual presence of a pathogen” (54). Fat storage in response to cues from the external environment presumably evolved in mammals as a mechanism for survival in environments with fluctuating food availability (63). In the temperate zone, for example, late summer and autumn feature plentiful food availability, during which humans likely encountered plant foods like berries with high levels of free sugars as well as food animals with large saturated fat stores. Consumption of intermittently available, energy-rich foods presumably promoted fat storage and increased survival through periods of low food availability. High levels of dietary saturated fat (64–66) and a high glycemic load (67, 68) have indeed been proposed to act as gene modulators that stimulate fat storage specifically around the belly (Figure 3). Today, the modern Western diet promotes continuous, year-round accumulation of visceral fat by providing excessively high levels of saturated fat (64–66) as well as of free sugars and quick-burning starches [high glycemic load (67, 68)], and thereby produces an exaggerated version of the body’s evolved response to intermittent times of ample energy supply (54).

One mechanistic link between the extent of energy storage and the strength of the immune response lies in the fact that visceral fat cells produce hormones (cytokines) that activate inflammation (51, 52, 69). It is thus not simply overall obesity, but rather the specific distribution of weight gain along a person’s waist that is related to chronic diseases and disorders (54, 68). A recent study (68) demonstrated that people can put on increased levels of belly fat even when consuming overall fewer calories than their bodies are burning, highlighting the importance of diet composition and quality in addition to total calories consumed. While obesity thus triggers excessive inflammation, the ability to fight infections is, unfortunately, also impaired by obesity (70).

### An Excessively High Ratio of Omega-6 to Omega-3 Polyunsaturated Fatty Acids Promotes Inflammation

Dietary omega-3 and omega-6 fatty acids are converted in the human body to hormones that regulate the immune system; omega-6-derived hormones typically stimulate the immune response and omega-3-derived hormones are anti-inflammatory [(66, 71, 72), see also Ref. (25, 59, 73, 74)]. Simopoulos (25, 59, 60) has stressed that a balanced ratio of dietary omega-6 to omega-3 oils should be lower than 10:1, and maybe as low as 2:1 or 1:1, and that the modern Western diet provides a highly unbalanced ratio exceeding 10:1, in large part due to the very high ratios occurring in mass-produced vegetable oils, such as corn, sunflower, soybean, and cottonseed oil. The high omega-6 to omega-3 fatty acid ratio typical of intensively reared beef likely results from the corn used as feed (75). Therefore, both species used (such as the plant species used as feed for livestock) and food production conditions affect nutritional quality. Other oils (such as canola oil) have more balanced ratios, and yet others (such as linseed oil) are high in omega-3 fatty acids (25). The anti-inflammatory traditional Mediterranean diet derives the majority of its fat content from the monounsaturated olive oil that contains neither omega-6 nor omega-3 fatty acids (45, 76). This diet’s anti-inflammatory properties also stem from a plethora of anti-inflammatory natural products contained in virgin olive oil and other components of the diet (76).

### A High Ratio of Oxidants to Antioxidants Promotes Inflammation

Both oxidants and antioxidants also serve as gene regulators of the human immune system; excessive levels of unopposed oxidants in the body increase the risk for inflammation-related chronic diseases (37–39). The balance of oxidants and antioxidants in the human body is affected not only by the modern Western diet, but also by physical inactivity and other factors related to lifestyle and/or environment. While important antioxidants must be acquired through diet, others (antioxidant enzymes) are produced in the body itself in response to oxidant triggers generated by muscles during physical activity (77).

It appears that humans are currently experiencing an unfavorable combination, where the modern environment and lifestyle produce excessively high levels of oxidants in the body, while the Western diet is deficient in antioxidants that detoxify oxidants. Although high-dose antioxidants are strongly promoted by supplement manufacturers, they may not have the desired effects; while a whole-food-based diet like the Mediterranean diet substantially lowered the risk of chronic diseases and disorders, single high-dose supplements did not – and even produced adverse outcomes (78–80). The available evidence is consistent with the view that whole foods contain a plethora of modulators that target multiple components of regulatory networks (37), which appears to be more effective in suppressing excessive responses, while simultaneously avoiding undesirable side effects.

Moreover, while physical inactivity lowers the levels of internally produced antioxidants (17, 77, 81), a host of other lifestyle factors actively generate oxidants in the body. Recent evidence suggests that the latter factors, as related to lifestyle and/or environment, include psychological stress, too little sleep, pollution and toxins, smoking, drugs, and excessive drinking [(22, 81–83); see also Ref. (84, 85)]. In conclusion, a combination of moderate physical exercise, balanced antioxidant intake from food, and attention to other lifestyle and/or environmental factors is recommended to promote wellness [see Ref. (23) for such a scenario for the case of cardiovascular disease].

### Living Organisms Are What They Eat: Insights and Solutions from an Evolutionary Perspective

The regulation of body function by dietary cues brings to mind the old adage that “you are what you eat.” The regulation of body form and function in response to environmental cues apparently allows living organisms to take advantage of opportunities in the environment and to protect themselves against environmental threats. While body composition and function of humans and their food animals is modulated by diet, the composition of crops is modulated by water, nutrients, and other environmental variables (Figure 1). As stated above, it is therefore important to take all aspects of the growth environment into consideration when aiming to produce nutritionally balanced crops and livestock.

Initially, ancestral human diets presumably had high levels of antioxidants and omega-3 oils, causing the human immune response to evolve to function in the presence of such an immunosuppressive diet. Crop-species selection, crop-growing conditions, and food processing appear to have lowered the immunosuppressive quality of the human diet, and we propose that the modern Western diet can simply be viewed as having taken this approach to an extreme, detrimental level [see also Ref. (86)]. In comparing the nutritional quality of various crop varieties, such as early and modern corn cultivars, Robinson (86) states, “Ever since farmers first planted seeds 100,000 years ago, we have been unwittingly selecting for plants that are high in starch and sugar and low in vitamins, minerals, fiber, and antioxidants.”

We synthesize recent evidence that the nutritional features of the modern Western diet synergistically produce chronic inflammation due to excessive levels of omega-6 oils, saturated fat, sugars, and starches as well as deficiency in micronutrients and antioxidants [see Ref. (18, 25)]. Conversely, the anti-inflammatory properties of a traditional Mediterranean diet are presumably the reason for its effectiveness in lowering the risk for chronic diseases and disorders [(76), see also Ref. (22)]. While beyond the scope of the present review, cell proliferation and growth are also modulated by environmental factors. For example, plant antioxidants slow down cell division and growth in herbivores (87), while antioxidant-deficient diets contribute to run-away cell proliferation and cancer (37).

The recent discoveries of the role of dietary factors and other lifestyle factors in programing our genes add a new dimension to the understanding of how our genes respond to change in the environment. It had long been known that changing environments precipitate genetic change over evolutionary time, and that differential gene activation during an individual’s development is involved, for example, in the formation of an eye versus a toe. Our paper draws attention to the emerging view that changes in gene activation lead to the changes in energy storage and immune response discussed above in direct response to environmental cues. The discovery that nutrients modulate the physiology of organisms prompted dramatic advances in the field of nutrition [for selected reviews, see Ref. (88–92)]. One major new area focuses on the modulation of genes by nutrients, which is the focus of the present overview. While this metabolic programing by dietary and other stimuli proceeds throughout the human lifespan, a particularly important period is fetal development, during which maternal diet impacts gene activation patterns and future disease risk in the offspring [(93–95); see Ref. (96) for a review on prenatal, postnatal, and trans-generational effects]. Conversely, a second major area of study of gene–nutrient interactions focuses on the involvement of genetic differences among individuals in the regulation of metabolism by nutrients. Relevant examples for the impact of human genetic background on the link between nutrition and health in the context of the present overview include genetic differences in the ability to digest sugars or starch (91, 97) and in the ability to convert dietary precursor omega-3 fatty acids to the critically important forms EPA and DHA (98–100), as well as the involvement of a genetic predisposition for a pro-inflammatory state (88).

## The Impact of Crop Growing and Livestock Rearing Conditions on Human Health and Environmental Outcomes

### Principal Connections

**Figure 4:**
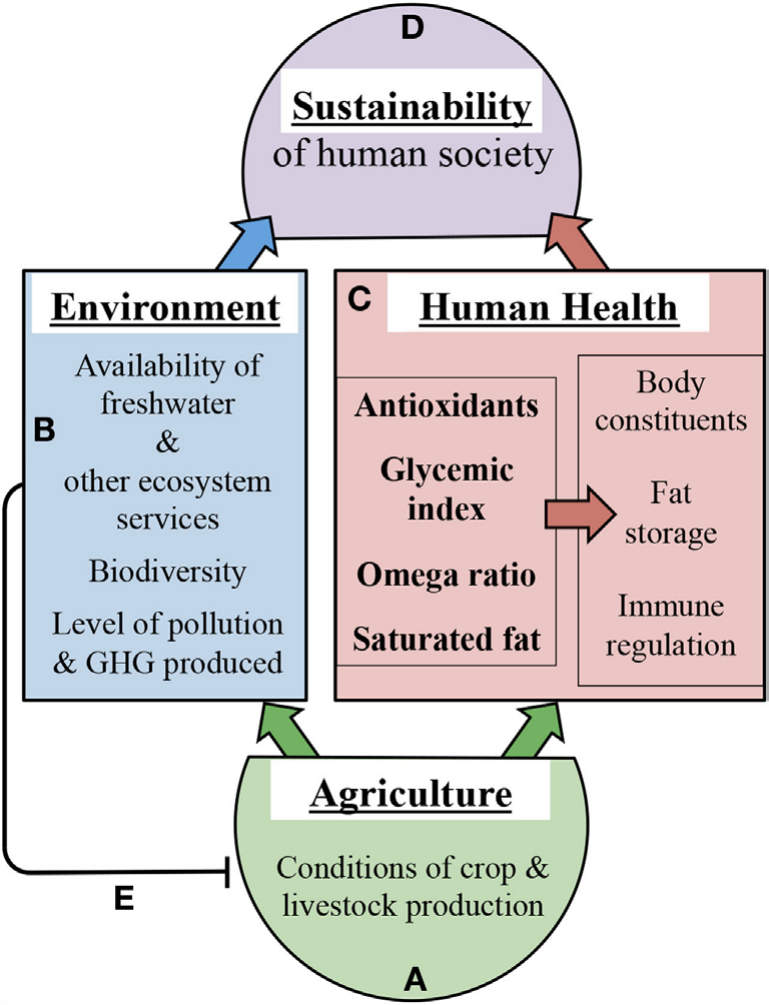
**Schematic depiction of selected examples of how agriculture (A) affects environment (B), human health (C), and thereby sustainability outcomes (D)**. The left box in **(C)** depicts crop and livestock composition with respect to the four selected nutritional features listed in Table 1 that are highly dependent on production conditions. The right box in **(C)** features human health outcomes impacted by crop and livestock composition. **(B)** features environmental outcomes impacted by crop and livestock production conditions. Line **(E)** depicts the negative feedback of intensive methods in modern agriculture on the health of agricultural systems and future food production. GHG, greenhouse gas. The links depicted here are intended to serve as examples rather than as an exhaustive list.

Figure 4 depicts selected mechanistic links between agricultural approaches and health outcomes on one hand or environmental outcomes on the other hand (Figures 4A–C). Our illustrative examples of such links focus on the impact of growth conditions on crop and livestock nutritional composition and are not intended to provide an exhaustive list of all factors involved. Additional factors linking human health to environmental sustainability include not only infectious microorganisms as stated above (16) but also a host of beneficial microbes belonging to the human microbiome (see below). Moreover, factors further contributing to nutritional imbalances include (i) the genetic background of crop species/varieties (101, 102) as well as that of the human consumer (91, 97–100), and (ii) food processing (103, 104), food availability (105), and dietary preferences (19). Improvements of food processing approaches as well as of equitable access to nutritious food for all members of society are clearly needed. Furthermore, improvements in agricultural approaches may have the potential to make additional contributions as highlighted in the present review. Neff et al. (20) point out that, in the context of public health, more attention has thus far been paid to what Americans eat than how the food is grown.

Features of modern, intensive agricultural production include high spatial concentrations of single plant and animal species, reliance on high resource input, and high waste production that are detrimental to the environment. Resource inputs include water, fertilizer, pesticides, and antibiotics, animal feed, and fuel, while waste produced includes polluting chemicals, excess nutrients, greenhouse gases, and manure. Figure 4B presents a brief overview of links between modern, intensive agricultural practices and environmental degradation. In brief, modern, intensive agriculture currently relies on unsustainable freshwater supplies [irrigation accounts for 70% of freshwater withdrawals; (1)] and releases pesticides and antibiotics into the environment. Modern, intensive agricultural practices also cause disruption of nitrogen and phosphorus cycles through nutrient pollution from fertilizer runoff (1) and concentrated manure waste (106). Over-fertilization of water bodies causes problems such as algal blooms, in the wake of which oxygen levels in the water plummet, reducing fish yields (107). Furthermore, biodiversity losses due to deforestation, habitat destruction, and pollution are discussed as factors promoting human and wildlife disease (108, 109). As pointed out by Hough (110), effects of “biodiversity on human health include the diversity of the internal microbiome” (with microbiome defined as the entirety of the plethora of microbes living in and on a human host), and the positive association of greater microbial diversity with improved human health (111). In the context of the present review, it is noteworthy that the composition of the human gut microbiome is strongly affected by environmental factors, and that the modern diet and lifestyle reduces microbe diversity and its health benefits [for a selected review, see Ref. (112)]. Finally, the high antibiotic use in concentrated animal feeding operations has been linked to the emergence of antibiotic-resistant microbial strains (113).

By contributing to climate change and environmental degradation (1), intensive methods in modern agriculture damage the very systems upon which agriculture depends (Figure 4E). Agriculture contributes heavily to CO_2_ greenhouse gas emissions *via* fossil fuel use, deforestation that eliminates trees as significant CO_2_ sinks, and release of the greenhouse gas methane from grazing ruminants like cattle, to name the major contributors (1). The rising level of greenhouse gases contributes to climate change and weather extremes (114) that negatively impact agricultural yields (13, 14).

We posit that modern, intensive agricultural practices also impact the nutritional quality of the food produced (Figures 4A,C). Our synthesis of the literature suggests that these agricultural production methods yield crops and livestock deficient in antioxidants and excessively high in glycemic index, in omega-6 to omega-3 ratios, and in saturated fat, all of which increase the risk of chronic diseases and disorders (Figures 3 and 4). Concentrated animal feeding operations, where animals are physically inactive and subject to grain feeding, produce meat of poor nutritional quality, i.e., with a high saturated fat content and high omega-6 to omega-3 fatty acid ratios (25, 115, 116), which presumably is harmful for human health (Figures 3 and 4). We propose that the impact of modern, intensive agriculture on human health and environment precludes achieving sustainability, and that a redesigned agriculture has the potential to achieve this goal (Figure 4D).

### Impact of Environmental Stress on Plant Antioxidant Content and Crop Yield

**Figure 5:**
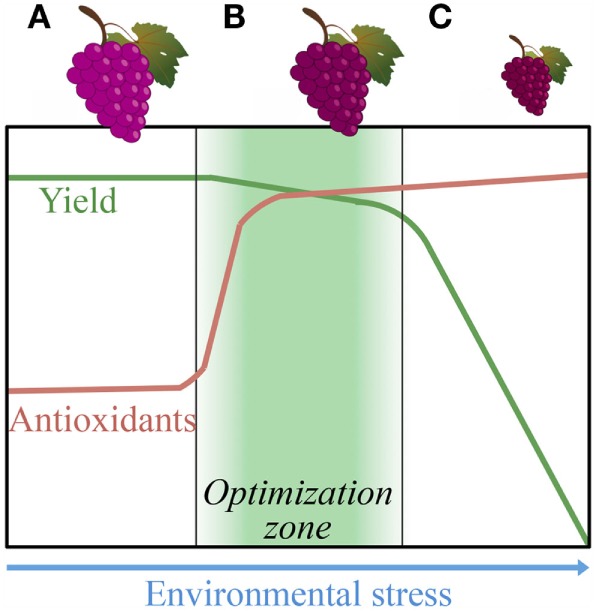
**Schematic depiction of the effect of environmental stress, such as decreasing water availability in the soil, on plant antioxidant content and crop biomass accumulation/yield**. The grape berry bunches across the top symbolize the effect of **(A)** ample irrigation, **(B)** limited irrigation, and **(C)** severe drought. The shaded area under **(B)** represents the area of co-optimization for the combination of optimal antioxidant content combined with optimal crop yield. The depicted relationships are only intended to highlight the concept and specifics (starting levels of antioxidants and degree of increase in response to stress) should be expected to vary among plant organs (leaves, fruit, seed) and by antioxidant type.

Modern, industrial-scale crop production has increasingly centered on monocultures of crop cultivars (elite cultivars) selected *via* intensive breeding programs and grown under environmental conditions that produce large plant or fruit size, uniform appearance, and durability (117). Selection using the latter criteria inadvertently reduced (i) nutritional quality and flavor (117–120) as well as (ii) plant resistance to drought and other environmental stressors (102). Growth of crops with high inputs of water, fertilizer, and pesticides apparently lowers nutritional quality (e.g., lowers antioxidant levels). In fact, the U.S. Department of Agriculture reported a decline in various nutrients (including the antioxidant vitamin C) in 43 crops between 1950 and 1999 (118). Figure 5 depicts our proposal of principal conceptual relationships among cultivation conditions, crop nutritional quality, and crop yield, and the potential feasibility of producing large quantities (high yields) of food with superior nutritional quality.

Plants synthesize antioxidants as well as additional plant chemicals (termed phytochemicals in a nutrition context) for their defense against oxidants produced in their own tissues under limited water supply and other environmental challenges, such as pests, pathogens, extreme temperatures, and limited soil nutrients (121, 122). Plants grown with ample irrigation and pesticide application would, therefore, be expected to produce fewer antioxidants and other defense chemicals. At the other extreme, severe environmental stress would arrest plant growth, and cause crop yield to plummet (Figure 5). We propose that the goal should be to explore a possible co-optimization zone (shaded area in Figure 5), where moderate environmental stress may trigger antioxidant accumulation without significantly cutting into crop yield (cf. Figure 5). Proof-of-concept that such a zone may indeed exist comes from practices used for growing grapevines. Winegrowers long noticed that high water input to their grapevines results in the production of less flavorful grapes as a result of low levels of plant defense chemicals [see Ref. (123)]. A rich bouquet is conferred to wine by a multitude of phytochemicals, many of which are potent antioxidants. Winegrowers can co-optimize grape yield and grape flavor (where flavor also indicates nutritional quality) by using precision irrigation, whereby water supply to the grapevines is limited to enhance flavor/antioxidant content, without cutting significantly into grape yield [Figure 5 (123), see also Ref. (124)]. As is the case for grape color and flavor, intense coloration and rich flavor can also serve as indicators of high nutritional quality for other foods, such as fish (125).

On the basis of this example, we propose that human society may not have to choose between food quantity and nutritional quality. This example is also an illustration of the profound effect of the environment on the composition of living organisms, including plants. Winegrowers have thus provided an inspiring example for the feasibility of successful co-optimization of crop yield and crop nutritional quality, while preserving water resources and making the crop more tolerant of fluctuations in environmental conditions, which becomes increasingly important in the context of climate change. Use of densely planted pruned orchards has also been employed to increase fruit yield and limit resource inputs for apples and avocado (126, 127).

While the above examples illustrate the impact of growing conditions, novel breeding efforts also hold promise. Yadav (128) crossed an extensively bred high-yielding but drought-sensitive elite line of millet with a low-yielding but drought resistant, locally adapted millet line and obtained some crosses that combine the desirable traits of both parents in being both high-yielding and resistant to drought, i.e., able to survive and thrive with low water inputs. In general terms, it has been suggested that sustainable agricultural practices could be modeled after mature natural ecosystems and operate as *agroecosystems* (129). Natural ecosystems become established along an S-curve of growth (130), where the initial phase features single, or a few, species (as is the case for the monocultures used in modern, intensive agriculture) whose growth and survival is driven by high levels of external inputs (129). Over time, ecosystems reach a state in which species diversity and interactions are high and few external inputs are needed. In contrast, modern, intensive agriculture typically “force[s] the growth process to start over” every year (129). Whereas modern, intensive systems thus remain at the base of the ecosystem growth curve and are dependent on continuous large external inputs (of, e.g., water, fertilizers, and animal feed), agricultural systems designed after the principles of mature ecological systems at the top of the growth curve could become more self-sustaining (129).

These new insights about health and agriculture suggest that it is both necessary and feasible to simultaneously protect against human disease by improving food nutritional quality and to protect future food production by preventing environmental degradation. In the context of sustainability, one could argue that protection of food production is required to meet the needs of future generations, while improvement of food nutritional quality is needed to meet the needs of both present and future generations.

## Application of Novel Mechanistic Insights toward Co-Optimizing Human Health, Environmental Sustainability, and the Ability to Feed the World Population

**Figure 6:**
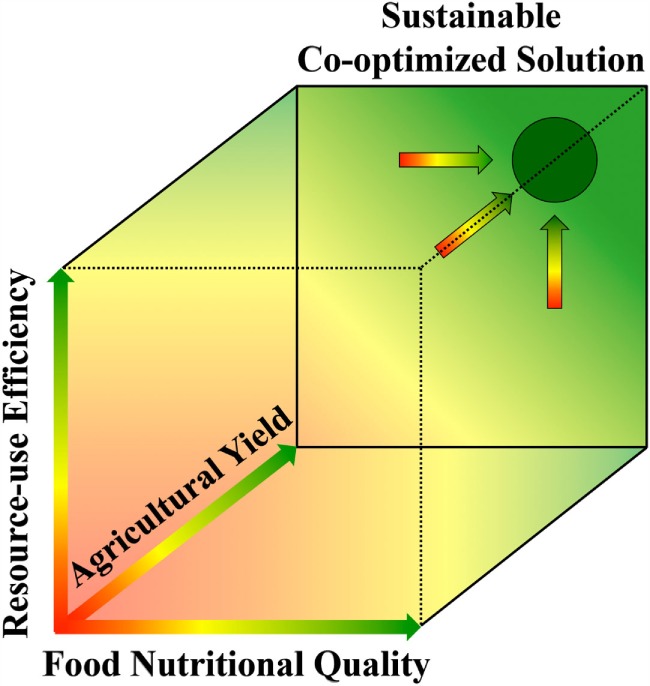
**Three-dimensional matrix with three axes representing the three parameters (*x*-axis) food nutritional quality (as linked to human health), (*y*-axis) crop resource-use efficiency (as linked to environmental health), and (*z*-axis) agricultural yield (as linked to social equity through the ability to serve the entire human population)**. The purple circle represents high values for all three parameters, thus symbolizing co-optimization of these three parameters *via* sustainable agricultural approaches.

The approach of searching for sustainable solutions at the interface between human health and environment is in good agreement with benefits of multiple essential products and values (collectively termed ecosystem services) provided to humans by the environment [for a review, see Ref. (131)]. This evaluation of ecosystem services shows that independent maximization of only a single parameter leads to undesirable outcomes by leaving other key needs unaddressed, whereas co-optimization of all relevant parameters in conjunction is needed to identify solutions that offer true sustainability (131). In their study on beef production, Dawson et al. (115) point to the “danger and inadequacy of considering each parameter in isolation” and evaluate multiple factors in a two-dimensional matrix addressing both meat quality and environmental gains. Figure 6 is inspired by the general approach taken by Dawson et al. (115) and extends the principle of co-optimization to a three-dimensional matrix for simultaneous optimization of human health (*via* food nutritional quality), environmental resilience (*via* resource-use efficiency), and social equity (*via* high agricultural yields). Figure 6 also describes our vision that co-optimization may allow single, or a few, optimal solutions for a given place and time to float to the surface, while aiding in elimination of alternatives that do not address all key parameters. While there is likely an optimal solution for a given place and time, sustainable solutions should be expected to differ by local context, including variations in physical environment as well as socioeconomic and geopolitical factors.

In conclusion, we propose that redesign of agricultural practices has the potential to contribute to the production of food that (i) is nutritious and fosters optimal human physical and mental well-being, (ii) is resource-efficient and thus environmentally sustainable, with (iii) optimal agricultural yields to support the entire human population and thereby promote additional dimensions of sustainability such as social stability and peace. Thus far, human society has made great strides in optimizing food quantity (132), i.e., the production of food biomass and calorie content, as well as features such as transportability, shelf life, and profit margins. The recent discovery of the power of the environment (including diet) in programing body composition and function of humans and their food plants and animals now needs to be put into action to produce health-promoting, nutritious food in sufficient quantity to equitably serve the growing global human population in a sustainable way (see Figure 6).

We argue that each of the three axes in Figure 6 has concrete ties to long-term economic benefit. Benefits of ecosystem services derived from a sustainable, resilient environment have been recognized (133). The specific economic benefits of improving human health through lifestyle management should go beyond the obvious benefits of reduced health-care costs to potential large gains in workforce productivity derived from improved mental health and acuity (17, 36, 134, 135). Economic benefits of equitable access of the entire human population to nutritious food presumably include not only increased workforce productivity, but also the potential to counteract violent conflict and enhance societal stability. From this perspective, agricultural practices become an important target for policy-making, and eliminating chronic diseases and disorders can become a powerful embodiment of the otherwise less tangible idea of sustainability.

## Co-Optimization in Action

What does a diet co-optimized on the basis of resource-use, nutritional quality, and the ability to feed the world population look like? Tilman and Clark (12) compare several diets that confer health benefits and lower environmental costs compared to current modern, intensive agricultural practice: vegetarian diets, fish and seafood-based (pescetarian) diets, and the Mediterranean diet. However, implementation of each presents challenges; the Mediterranean biome occupies only a small region of the planet, global fish populations are declining, and vegetarian diets vary dramatically in their composition and health impact.

We suggest that the four key dietary factors highlighted in the present paper could serve to design a variety of healthful diets based on different foods and featuring different proportions of carbohydrates, fats, and protein. Identification of the underlying features that matter for health vastly expands the number of viable nutritious diet options around the globe to accommodate local climatic, topological, cultural, and economic contexts, thereby offering unprecedented flexibility that can support equity at various geographic scales. Understanding what matters for health underscores the recommendation by authors like Foley et al. (1) and Lichtfouse (136) to set aside ideological differences regarding agricultural practices and utilize all available approaches to increase the sustainable production of health-promoting foods. In the following, we offer specific suggestions on how the understanding of the four key nutritional features listed in Table 1 can guide optimization of vegetarian diets, fish-based diets, or any local diet. Once again, it should be noted that the revised agricultural approaches advocated here should be implemented in conjunction with revisions in food processing and with improvements in equitable access to nutritious food.

### Vegetarian Diets

Vegetarian diets vary widely in their composition and need to be carefully designed to meet the four key nutritional requirements listed in Table 1. McEvoy et al. (137) conclude that, “restrictive and monotonous vegetarian diets may result in nutrient deficiencies with deleterious effects on health … appropriate advice is important to ensure a vegetarian diet is nutritionally adequate.” Modern vegetarian diets, and especially vegan diets, are frequently depleted in long-chain omega-3 fatty acids as well as micronutrients and antioxidant minerals (138). While certain plant-based foods, like some nuts and seeds (linseed), contain high levels of omega-3 oils, the omega-3 fatty acid present in plants is a precursor molecule that needs to be converted to the long-chain omega-3 fatty acids DHA and eicosapentaenoic acid (EPA). It is the latter two omega-3 fatty acids that are required by humans as body constituents and precursors for immune-system-controlling hormones (Figure 3). Humans do not efficiently convert the precursor molecule found in plant foods to DHA and EPA, and, as stated above, there are, furthermore, individual (presumably genetic) differences in how inefficient this conversion is (98–100). Supplementation with fish oils or algal oils as well as breeding or engineering of crops for higher levels of omega-3 oils may make a contribution to ameliorating vegetarian diets. However, just as for antioxidant supplements (139), concerns have been raised about negative health outcomes associated with the use of omega-3 supplements (140) versus whole foods containing these nutrients.

As outlined above, there are several lines of evidence suggesting that the antioxidant content of our foods has gone down as a result of modern agricultural practices (86, 102, 118). To enhance crop antioxidant content *via* revised agricultural approaches, precision irrigation could be implemented to increase crop antioxidant content without sacrificing yield (see Figures 5 and 6). In addition, the use of currently underutilized vegetable (141, 142) and grain species should be expanded. For example, the grains quinoa and teff have a high antioxidant content, balanced omega-6 to omega-3 ratios, a low glycemic index, a high tolerance of environmental stresses, and are also replete in essential amino acids (143, 144). These grain species can be grown with high yields under low inputs of water, fertilizers, and pesticides. Additional opportunities for the improvement of the nutritional value of vegetarian diets include attention to the glycemic index of plant-based food. The nutritional quality of potato can be ameliorated by decreasing quick-burning starch (like the highly branched amylopectin that is digested to glucose very quickly) and increasing slow-burning starch (like the unbranched amylose that is digested to glucose less quickly) (145). Moreover, the processing of whole grain to white flour, which removes vital micronutrients and elevates the glycemic index, should be minimized. It should, furthermore, be noted that the digestibility of potato starch is also affected by food preparation (146) and by the non-carbohydrate components of a meal (147).

### Pescetarian Diets

Pescetarian diets provide high levels of omega-3 fish oils only if the fish has consumed microorganisms (typically cold-water algae) that synthesize these omega-3 oils – since fish, like humans, “are what they eat” (29) and must consume organisms that produce long-chain omega-3 fatty acids. Fish farms should thus be designed with attention to providing food that contains long-chain omega-3 oils, as is done on the model farm Veta La Palma in Spain that sustainably produces high yields of nutritious, extraordinarily flavorful fish without external net inputs other than sunlight supporting the growth of photosynthetic algae as fish food (125). Efforts are also underway to develop EPA-rich plant-based sources of fish feed for aquaculture (148).

### The Traditional Mediterranean Diet

The traditional Mediterranean diet, rich in vegetables and fruit, whole grain, and seafood, with small contributions of lean meat (45), is a model diet that provides an example for how all four dietary features highlighted here can be accommodated. While the traditional Mediterranean diet was used in clinical trials that provided evidence of a concrete health benefit, it is likely that the same health benefits are offered by other traditional diets with similar features, i.e., replete in antioxidants and omega-3 oils with a low glycemic index/load and low saturated fat levels. McEvoy et al. (136) observe, “prudent plant-based dietary patterns which also allow small intakes of red meat, fish and dairy products have demonstrated significant improvements in health status.”

While meat from inactive animals raised with imbalanced supplemental feed is high in saturated and omega-6 fatty acids, free-ranging, grass-fed meat is typically low in saturated fat with a balanced omega-6 to omega-3 fatty acid ratio and rich in vitamins and antioxidant minerals (75, 115, 116, 149). From a health perspective, exclusion of meat from the human diet thus does not appear to be necessary. From the perspective of social responsibility, it has been pointed out that (i) in the absence of substantial irrigation, arid shrub-land and grassland areas can only produce food via rearing of grazing ruminants (1, 150, 151) and that (ii) rearing livestock is not only an important source of nutrition but also of economic stability for developing regions (7, 152, 153). Although a shift from animal-based to plant-based foods is advocated by many authors for reasons of environmental sustainability (1, 154–156), models for the sustainable production of animal-source food have also been described [e.g., Ref. (7, 125, 149, 157, 158)].

## Conclusion

We identified recent breakthroughs in the recognition of genetic programs that inextricably link form and function of all living organisms (humans and their food plants and animals alike) to the environment; we identified saturated fat, polyunsaturated omega-6 and omega-3 oils, carbohydrates (glycemic load), and antioxidants as key diet-related modulators of these genetic programs, thus providing options for designing health-promoting diets for a range of specific contexts; we also identified additional, synergistically acting lifestyle-related factors that modulate the same genetic programs. We specified changes to agricultural practices that promise to simultaneously benefit environmental sustainability and human health around the globe.

Improving physical and mental health by adjusting human diet (and lifestyle) promises to provide benefits to individuals, families, and local and world communities. These benefits, combined with the prospect of increased workforce productivity, can provide concrete incentives to governments, businesses, and policy makers to provide equitable access to nutritious foods for all members of society. Sustainability can be viewed as including healthy human populations, a resilient environment and robust current and future agricultural productivity, and minimization of social strife, which all translates into economical and quality-of-life value to human society. The potent gene-programing effect of the human environment emphasizes the threat to human health presented by the current environment, while also illuminating unprecedented potential gains in quality-of-life fostered by a future environment designed to optimize human wellness. Such a vision of human wellness, fitness, and mental acuity supported by a sustainable environment can serve as an inspiring image of what sustainability has to offer to individuals and human society at large.

## Author Contributions

MA and RA conceived the project with contributions from BD-A. MA and BD-A conducted data search and analysis with contributions from CW and RA. MA drafted the work with input from BD-A and contributions from RA, CW to draft revision.

## Conflict of Interest Statement

The authors declare that the research was conducted in the absence of any commercial or financial relationships that could be construed as a potential conflict of interest.
